# Genomic epidemiology and the evolution of erm(B)-mediated macrolide resistance in Campylobacter

**DOI:** 10.1099/mgen.0.001528

**Published:** 2025-10-08

**Authors:** Fen Gao, Frances M. Colles, Seungwon Ko, Jiayuan Luo, Samuel K. Sheppard, Min Chen

**Affiliations:** 1 Department of Microbiology, Shanghai Municipal Center for Disease Control and Prevention, Shanghai, PR China; 2 Department of Biology, University of Oxford, Oxford, UK

**Keywords:** accessory elements, antimicrobial resistance, *Campylobacter*, *erm*(B), macrolides

## Abstract

*Campylobacter* is a major foodborne bacterial pathogen that has become increasingly resistant to clinically important antimicrobials. Of particular concern is the emergence of *erm*(B)-mediated macrolide resistance, which has been increasingly documented across *Campylobacter* isolates from diverse ecological reservoirs. In this study, we investigated the genomic characteristics and epidemiology of *erm*(B)-carrying clinical *Campylobacter* isolates from Shanghai, alongside a globally representative dataset of all publicly available strains. Among clinical isolates obtained from a diarrhoeal outpatient surveillance programme between 2020 and 2023 in Shanghai, China, 16% (80/500) were erythromycin-resistant, with 23.8% (19/80) testing positive for *erm*(B). The genomes of these isolates were sequenced to identify *erm*(B) gene alleles. Phylogenetic analyses, pairwise comparisons of core and accessory genomes and examination of shared alleles revealed horizontal gene transfer as the predominant mechanism driving the transmission of *erm*(B) between isolates from various sources. Poultry was identified as a key reservoir for human infections caused by *erm*(B)-positive *Campylobacter* isolates. Comparative pangenome analyses of *erm*(B)-positive and negative isolates identified multiple accessory elements associated with *erm*(B) acquisition, among which the IS607 family transposon-associated *tnpB* gene exhibited sequence and structural homology to functional progenitors of CRISPR-Cas nucleases. These findings expand our understanding of the epidemiology of *erm*(B)-mediated macrolide resistance in *Campylobacter* and underscore the urgent need for enhanced antimicrobial stewardship in poultry production and targeted surveillance programmes to curb the spread of resistance.

**Impact Statement**

The global prevalence of the macrolide resistance gene *erm*(B) in *Campylobacter* represents a significant public health threat. Although increasing reports of *erm*(B)-mediated resistance have been documented, the global dissemination of *erm*(B)-carrying lineages in human infections and other reservoirs remains unclear. In this study, we investigated the prevalence of the *erm*(B) gene in clinical isolates, identifying 80 erythromycin-resistant isolates through *in vitro* antimicrobial susceptibility testing, 19 of which were *erm*(B)-positive. Genomic analysis of clinical isolates and a global dataset revealed that poultry serves as a critical reservoir for the *erm*(B) gene, acting as a primary source of human infections. Horizontal gene transfer plays a key role in the acquisition and spread of the *erm*(B) gene across diverse genetic backgrounds. These findings underscore the need for a One Health approach, emphasizing collaboration among public health, veterinary and environmental sectors to develop integrated strategies to curb the spread of antibiotic resistance.

## Data Summary

Whole-genome sequencing data in this study are deposited in the NCBI GenBank database under the BioProject PRJNA1207921 (https://www.ncbi.nlm.nih.gov/bioproject/1207921).

## Introduction

*Campylobacter* are among the most common causes of bacterial foodborne gastroenteritis worldwide [1,2]. The increasing prevalence of antimicrobial resistance (AMR) in the two most common pathogenic species, *Campylobacter jejuni* and *Campylobacter coli*, is now considered a serious threat to public health [3,4]. In recent years, increasing resistance to clinically important antibiotics used to treat severe *Campylobacter* infections, especially macrolides such as erythromycin, has limited the treatment options of campylobacteriosis [1,5]. Antimicrobial-resistant *Campylobacter* are more common in China than in some high-income countries [6,7]. For example, in structured sampling studies in Shanghai between 2012 and 2019, 2.5% and 59% of *C. jejuni* and *C. coli*, respectively, were resistant to erythromycin [2,7]. This is between three and six times higher erythromycin resistance than European studies (0.7% for *C. jejuni* and 10% for *C. coli*) [2,7]. As antimicrobial use in food animals is a major driver of AMR, the historical extensive use of antimicrobials in China, particularly macrolides, which were among the most commonly used classes in food animal production, may have contributed to the higher levels of erythromycin resistance observed in *Campylobacter* isolates [8].

There are multiple mechanisms underlying macrolide resistance in *Campylobacter*, including mutations in 23S rRNA and ribosomal proteins L4 and L22, the CmeABC efflux system and the *erm*(B) gene [9]. The *erm*(B) gene encodes a 23S rRNA methyltransferase which confers high-level resistance to multiple antibiotic classes, including macrolides, lincosamides, streptogramin B and telithromycin [10,11]. Since its first discovery in *Campylobacter*, the *erm*(B) gene has been detected primarily in *C. coli* isolates from both animal and clinical sources in China and several other countries [10,12,15]. Investigation into the genetic environment of *erm*(B) has revealed that it is commonly present within multidrug resistance genomic islands (MDRGIs) [16]. This facilitates horizontal gene transfer (HGT) along with other antibiotic resistance genes among bacterial populations [17,18].

Epidemiological analyses have revealed a broad distribution of *erm*(B)-harbouring isolates and the proliferation of dominant lineages [10,17, 19]. Furthermore, the increasing availability of genomic data from structured surveillance has improved understanding of resistant lineages by inferring AMR in isolate genomes from different sources. However, tracking the global spread of important resistance genes, including *erm*(B), requires a deeper understanding of resistant *Campylobacter* populations. In this study, we analysed the AMR of 500 clinical isolates from a surveillance programme of outpatients with diarrhoea between 2020 and 2023 in Shanghai, China. The clinical *erm*(B)-carrying isolates were systematically compared with a global collection of isolates from various sources using comparative genomic approaches. Pangenome analyses identified the accessory genes and transposable elements (TEs) associated with the emergence of *erm*(B)-mediated resistance in *Campylobacter*.

## Methods

### *Campylobacter* isolates and whole-genome sequencing

A total of 500 *Campylobacter* isolates were collected between 2020 and 2023 by the Shanghai Municipal Center for Disease Control and Prevention (Shanghai CDC, China). The clinical strains were isolated from stool samples of patients with gastroenteritis symptoms admitted to sentinel hospitals across Shanghai. A 5 g fresh stool sample was obtained from each patient and sent to the laboratory for bacterial isolation. The isolation and identification of the isolates were conducted as previously described [1,7] using the filter-based method with *Campylobacter* isolation kits (ZC-CAMPY-001, Qingdao Sinova-HK Biotechnology Co., Ltd., Qingdao, China). Briefly, the faecal specimen was suspended in the enrichment broth provided in the kit and cultured at 42 °C for 24–48 h in a microaerobic environment (5% O_2_, 10% CO_2_ and 85% N_2_). Approximately 300 µl of the enriched medium was filtered through a 0.45 µm membrane filter onto a Columbia Agar supplemented with 5% defibrinated sheep blood (Shanghai Kemajia Microbiotech Co., Ltd., Shanghai, China). Single colonies resembling *Campylobacter* were sub-cultured for further testing, including identification using the MALDI-TOF-MS (bioMérieux, France) and PCR analysis.

Genomic DNA from the isolates was extracted using the QIAamp DNA mini kit (Qiagen, Germany) following the manufacturer’s instructions. All isolates in this study were screened for the *erm*(B) gene using PCR [20], and paired-end sequencing was performed on all isolates carrying the *erm*(B) gene using Illumina NovaSeq 6000 (Illumina, USA). The sequencing reads were assembled *de novo* using SPAdes [21]. The quality of the genome was assessed using CheckM v1.2.2 [22], ensuring that the contamination level was less than 5%. Assembled genomes of isolates carrying the *erm*(B) gene were archived in the PubMLST database (https://pubmlst.org) which runs using BIGSdb software (Table S1, available in the online Supplementary Material) [23,24]. Isolates from other countries and sources, with genomes archived in the PubMLST database (~80,000 *Campylobacter* genomes accessed on 27 August 2024), were screened for the presence of the *erm*(B) using the Gene Presence plugin. The sequence types (STs) and clonal complexes (CCs) were determined for 259 isolates including *erm*(B)-harbouring isolates collected in this study, clinical *erm*(B)-positive *C. coli* isolates [7] and 210 archived genomes containing the *erm*(B) gene using the PubMLST database [25]. The allelic variation of *erm*(B) was assigned using the PubMLST database.

### Antimicrobial susceptibility testing

Minimum inhibitory concentrations (MICs) were determined for the 500 *Campylobacter* isolates using the broth microdilution method and commercial kits for six chemically distinct classes of antimicrobial drugs in accordance with the manufacturer’s instructions (Shanghai Fosun Long March Medical Science Co., Ltd., Shanghai, China). These included aminoglycosides (gentamicin, GEN), macrolides (erythromycin, ERY), lincosamides (clindamycin, CLI), phenicols (florfenicol, FFN), quinolones (nalidixic acid, NAL) and tetracyclines (tetracycline, TET). Phenotypic resistance was interpreted according to the standard of the National Antimicrobial Resistance Monitoring System (NARMS) [26]. The breakpoint values for *C. jejuni* and *C. coli* were different for some drugs, including ≥1 µg ml^−1^ for CLI, ≥8 µg ml^−1^ for ERY and ≥2 µg ml^−1^ for TET for *C. jejuni* and ≥ 2 µg ml^−1^ for CLI, ≥16 µg ml^−1^ for ERY and ≥4 µg ml^−1^ for TET for *C. coli*, respectively. For other drugs, *C. jejuni* and *C. coli* have the same MICs: ≥8 µg ml^−1^ for FFN, ≥4 µg ml^−1^ for GEN and ≥32 µg ml^−1^ for NAL.

### *In silico* identification of putative AMR and virulence genes

AMR and virulence genes were identified using Abricate v1.0.1 (https://github.com/tseemann/abricate) based on the NCBI AMRFinder [27] and Virulence Factor Database (VFDB) [28], respectively. Positive hits were filtered using a minimum DNA identity of 70% and sequence coverage of 70%. Point mutations associated with AMR genes were detected using PointFinder [29].

### Core and accessory genome analyses

Genomes were annotated using Prokka v1.14.6 [30] and the pangenome was generated using PIRATE v1.0.5 [31]. This identified core genes present in 95% of the genomes and accessory genes present in at least one isolate. Orthologous genes were clustered using Markov cluster algorithm over a wide range of aa identity thresholds, including 45%, 50%, 60%, 70%, 80%, 90%, 95% and 98%. Core-genome variation between isolates was identified by calculating the pairwise average nt identity (ANI) using FastANI v1.33 [32]. The gene presence matrix generated by PIRATE was utilized to create a heatmap illustrating shared pairwise accessory-genome genes. A core-genome alignment was generated for all 259 *Campylobacter* isolates by concatenating gene sequences present in 95% of genomes, using a gene-by-gene approach [33]. A maximum-likelihood phylogeny was generated for the *Campylobacter* isolates using FastTree v2.1.11 [34] and visualized on Microreact [35] and iTOL [36], incorporating the AMR and VFDB profiles of each isolate.

### Comparative pangenome and protein analyses

*C. jejuni* and *C. coli* genomes from this study, and archived strains, were analysed to identify pangenome differences between *erm*(B)-positive and *erm*(B)-negative isolates. A total of 611 *C*. *jejuni* isolates from various countries and sources were chosen with up to 20 isolates per CC to maximize the representation of genetic diversity across the species. Similarly, 794 *C*. *coli* isolates were chosen including isolates from the 3 ancestral clades and hybrid CC828 and CC1150 lineages. Pangenome plots generated by Roary v3.13.0 [37] were used to determine the relationship between identified genes and number of genomes added. The pangenome-wide association analyses were performed using Scoary [38] by comparing the gene content of *erm*(B)-negative isolates with *erm*(B)-positive isolates for both *C. jejuni* (Table S2) and *C. coli* (Table S3). The gene presence/absence matrix was used to calculate gene prevalence, and accessory elements associated with *erm*(B) were identified (*P*-value=0.05). *In silico* sequence and structural analyses were conducted for TnpB. A homology search for TnpB was performed by blast comparison on the Protein Data Bank database server [39]. Alignment of multiple protein sequences was conducted using the COBALT program [40] and rendered by ESPript [41]. The 3D model of TnpB was constructed using AlphaFold 3 [42,43]. PyMOL software [44] was employed to represent the protein structure.

## Results

### Erythromycin resistance has increased considerably in *C. coli*

**Fig. 1.**
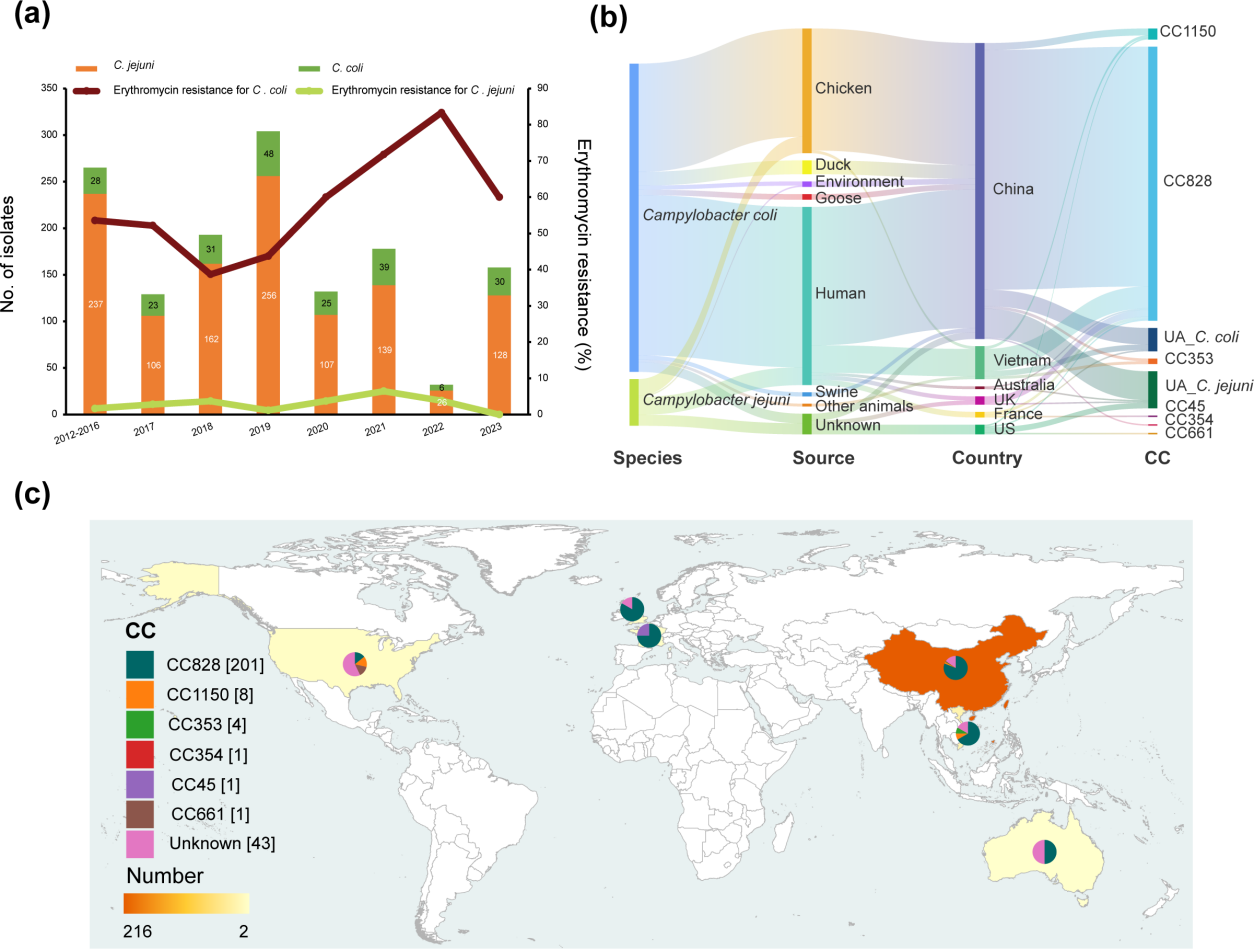
Putative erythromycin resistance in *Campylobacter* from China and global distribution of *erm*(B)-carrying isolates. (**a**) Number of *Campylobacter* isolates and percentage of *Campylobacter* isolates with macrolide (erythromycin) resistance in Shanghai, China (2012–2023). (**b**) Sankey diagram showing the species, sources, countries and CCs of global collection of *erm*(B)-carrying isolates. (**c**) Global distribution of 259 *erm*(B)-carrying isolates. The number of isolates in different geographical locations is shown by the shades of colour. The distribution of CCs in each country is indicated by pie charts with different colours representing different CCs.

A total of 500 clinical *Campylobacter* isolates, comprising 400 *C*. *jejuni* (80.0%, 400/500) and 100 *C*. *coli* (20%, 100/500), were collected from individual patients with diarrhoea across 32 hospitals in Shanghai between 2020 and 2023 (Fig. 1a). *C. jejuni* was the predominant species, and the proportion of *C. coli* had increased from 10.6% during 2012–2016 to 19.0% compared to a previous study [7].

Antimicrobial susceptibility testing identified 80 erythromycin-resistant strains, including 14 *C*. *jejuni* isolates and 66 *C*. *coli* isolates. A significant rise in erythromycin resistance was observed in *C. coli*, while resistance in *C. jejuni* remained relatively low as seen in previous studies (Fig. 1a). Overall, erythromycin resistance in *C. coli* was significantly higher than in *C. jejuni* (Fisher’s exact; *P*<0.001), which was consistent with previous studies [7,45]. Among the isolates, 3.8% (19/500) carried the *erm*(B) gene, with a marked predominance in *C. coli* (94.7%, 18/19). All *erm*(B)-positive isolates exhibited multidrug resistance and were resistant to at least three classes of antibiotics. The most common AMR profile was erythromycin (ERY) - nalidixic acid (NAL) - gentamicin (GEN) - tetracycline (TET) - clindamycin (CLI) (52.6%, 10/19). Notably, four isolates, including three *C*. *coli* and one *C*. *jejuni* isolates, displayed resistance to all tested antimicrobial agents.

### The *erm*(B) gene is globally disseminated in multiple lineage backgrounds

Nearly all of the *erm*(B)-positive isolates (18/19) were *C. coli*, with 17 assigned to the globally disseminated CC828 lineage (*n*=17). One isolate was not assigned to any CC. The single *erm*(B)-positive *C. jejuni* isolate was assigned to a novel ST, ST-14136 [46]. Multilocus sequence typing analyses assigned the 18 *C*. *coli* isolates into 13 STs within CC828, including 4 newly assigned STs in this study. Among these, ST-3753 was the most common (*n*=3), followed by ST-1145 (*n*=2), ST-825 (*n*=2) and ST-1586 (*n*=2). The *erm*(B)-positive *C. jejuni* isolate did not cluster within a known CC.

To gain insight into the genomic characteristics and epidemiology of local clinical *Campylobacter* isolates, we compared our clinical dataset with other *erm*(B)-positive isolates from various sources (Table S1). The full *erm*(B) dataset comprised 225 *C*. *coli* and 34 *C*. *jejuni* isolates, originating from humans (*n*=131), poultry (chicken, *n*=91; ducks, *n*=10; and geese, *n*=4), pig (*n*=3), the environment (*n*=4), other animals (*n*=2) and other sources (*n*=14) (Fig. 1b). Among the dataset, 83.4% of isolates (216/259) were obtained from China, comprising 101 from human, 89 from chicken and 26 from other sources (including 10 from ducks, 4 from environment, 4 from geese, etc.) (Fig. 1b, c). The remaining 43 isolates were collected from other countries, including 24 from Vietnam, 7 from the USA, 6 from the UK, 4 from France and 2 from Australia (Fig. 1b, c).

Significant diversity and structural variation were observed among genotypes of global *erm*(B)-positive isolates. A total of 79% of *C. jejuni* isolates (*n*=27) belonged to 25 diverse STs, none of which were assigned to any recognized CC. The remaining seven isolates were from lineages including CC353 (*n*=4), CC354 (*n*=1), CC661 (*n*=1) and CC45 (*n*=1). While CC45 is a host generalist lineage [47,49], the others are poultry-associated CCs frequently linked to human infections [50,52]. The sampling of CC353 isolates from both human and chicken sources is consistent with a common transmission route.

In contrast to *C. jejuni*, *C. coli* isolates primarily clustered within CC828 or CC1150, with only 16 out of 225 isolates not assigned to a CC. Overall, the 225 *C*. *coli* isolates represented 67 STs, with the most abundant being ST-1145 (*n*=37, 16.44%), followed by ST-872 (*n*=26, 11.56%) and ST-825 (*n*=24, 10.67%). Among these 67 STs, 56 belonged to 2 major CCs, ST-828 complex (*n*=201, 89.33%) and ST-1150 complex (*n*=8, 3.56%). These CCs belong to a single *C. coli* clade, which accounts for the majority of *C. coli* isolates found in human disease and agriculture, where extensive genetic recombination with * C. jejuni* has occurred [53,55].

### Genetic diversity and clustering of *erm*(B)-positive *Campylobacter* isolates

**Fig. 2.**
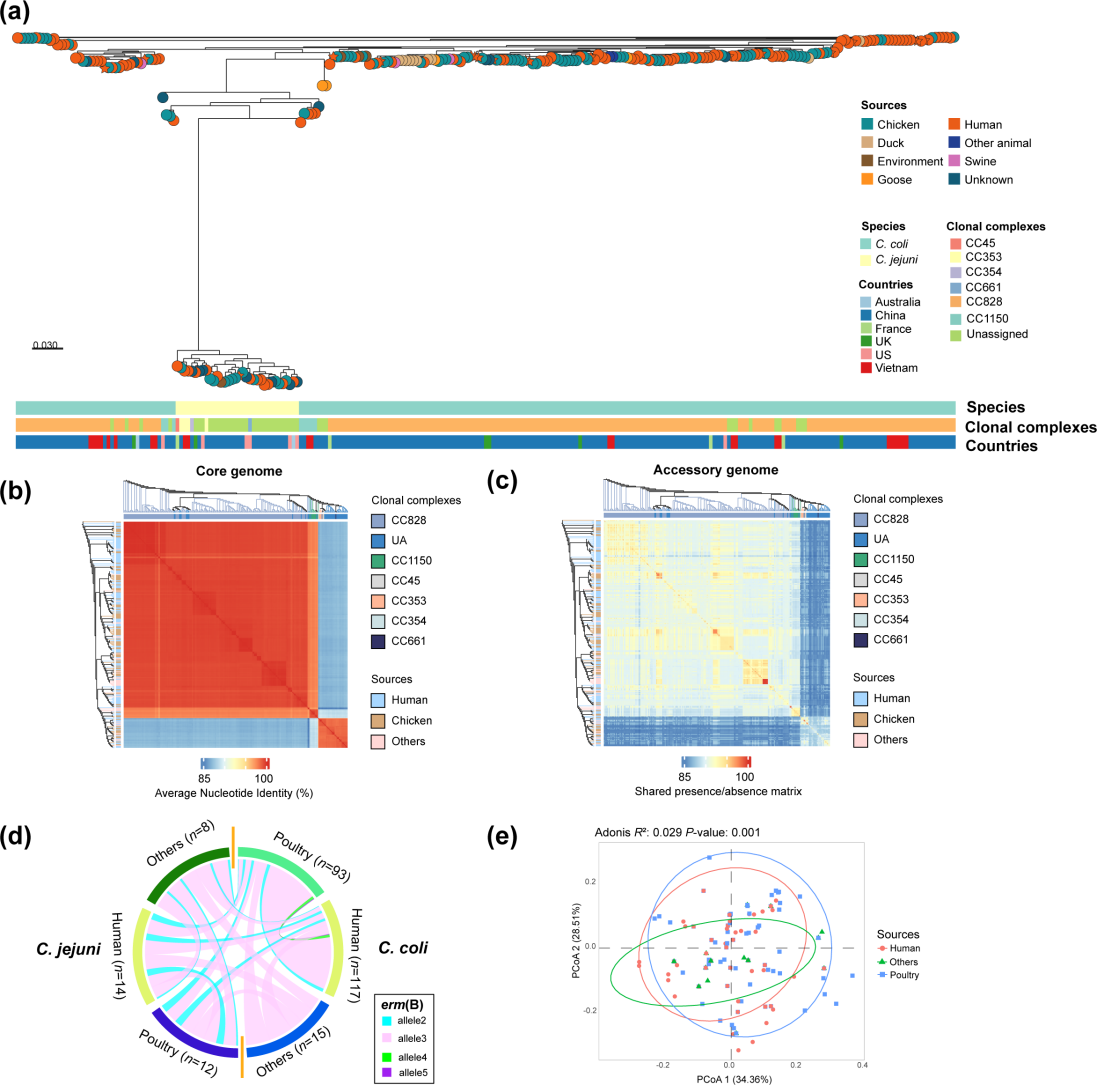
Genomic comparison of globally disseminated *erm*(B)-carrying *Campylobacter* isolates. (**a**) Phylogenetic tree of 259 *erm*(B)-carrying *Campylobacter* isolates. Colours indicate the species, sources, countries and CCs of the isolates. (**b**) Heatmap of pairwise ANI of all *Campylobacter* isolates ordered according to the phylogenetic tree; source and CC are indicated. Core genome similarity within species and CCs (*C. jejuni*) is clearly visible. (**c**) Heatmap of pairwise accessory genome similarity (gene presence/absence) among *Campylobacter* isolates ordered according to the phylogenetic tree; source and CC are indicated. (**d**) Distribution of *erm*(B) alleles among *Campylobacter* species and sources. Shared allelic sequences between isolates are indicated by joining lines, coloured differently for different alleles. (**e**) Principal coordinate analysis (PCoA) for antimicrobial resistance genes (ARGs) among *C. jejuni* and *C. coli* isolates from different sources, human, poultry and others. The x-axis accounts for 34.36% of the variance, while the y-axis accounts for 28.51% variance. Each dot represents an isolate, with different colours indicating different groupings.

A phylogenetic tree was constructed to better understand the genetic relatedness among *erm*(B)-positive isolates from China and other geographical regions and sources. This revealed distinct clustering patterns between *C. coli* and *C. jejuni*. Specifically, *C. jejuni* exhibited greater genetic diversity, while *C. coli* isolates tended to cluster within known CCs CC828 and CC1150. Overall, the isolates primarily clustered by CC rather than geographic origin or source (Fig. 2a). A detailed comparative analysis focusing on core genome similarity and shared accessory genes was further conducted (Fig. 2b, c). A total of 3,368 gene families were identified in 259 genomes using PIRATE [31]. These included 1,371 core gene clusters present in at least 95% of the isolates and 1,997 accessory genes. Pairwise comparison of core-genome ANI and shared accessory genes revealed a clear population structure (Fig. 2b, c). Clinical isolates were distributed among isolates from various sources on the phylogeny (Fig. 2a), suggesting shared transmission routes involving agriculture and human activity. No distinct geographical or source-based clustering was observed in either the core or accessory genomes. This is consistent with widespread transmission of *erm*(B)-positive isolates across sources and geographic boundaries. Allelic variation of the *erm*(B) gene was compared among *C. jejuni* and *C. coli* isolates (Fig. 2d). Four out of six *erm*(B) alleles were shared among *C. jejuni* and *C. coli* isolates from multiple sources. The most common allele, allele 3, was present in 23 *C*. *jejuni* and 185 * C*. *coli* isolates (Fig. 2d). The circulation of *erm*(B) alleles is consistent with gene pool spread into multiple genetic backgrounds through HGT.

### There was considerable variation in putative antimicrobial resistance and virulence genes among *erm*(B)-positive isolates

**Fig. 3.**
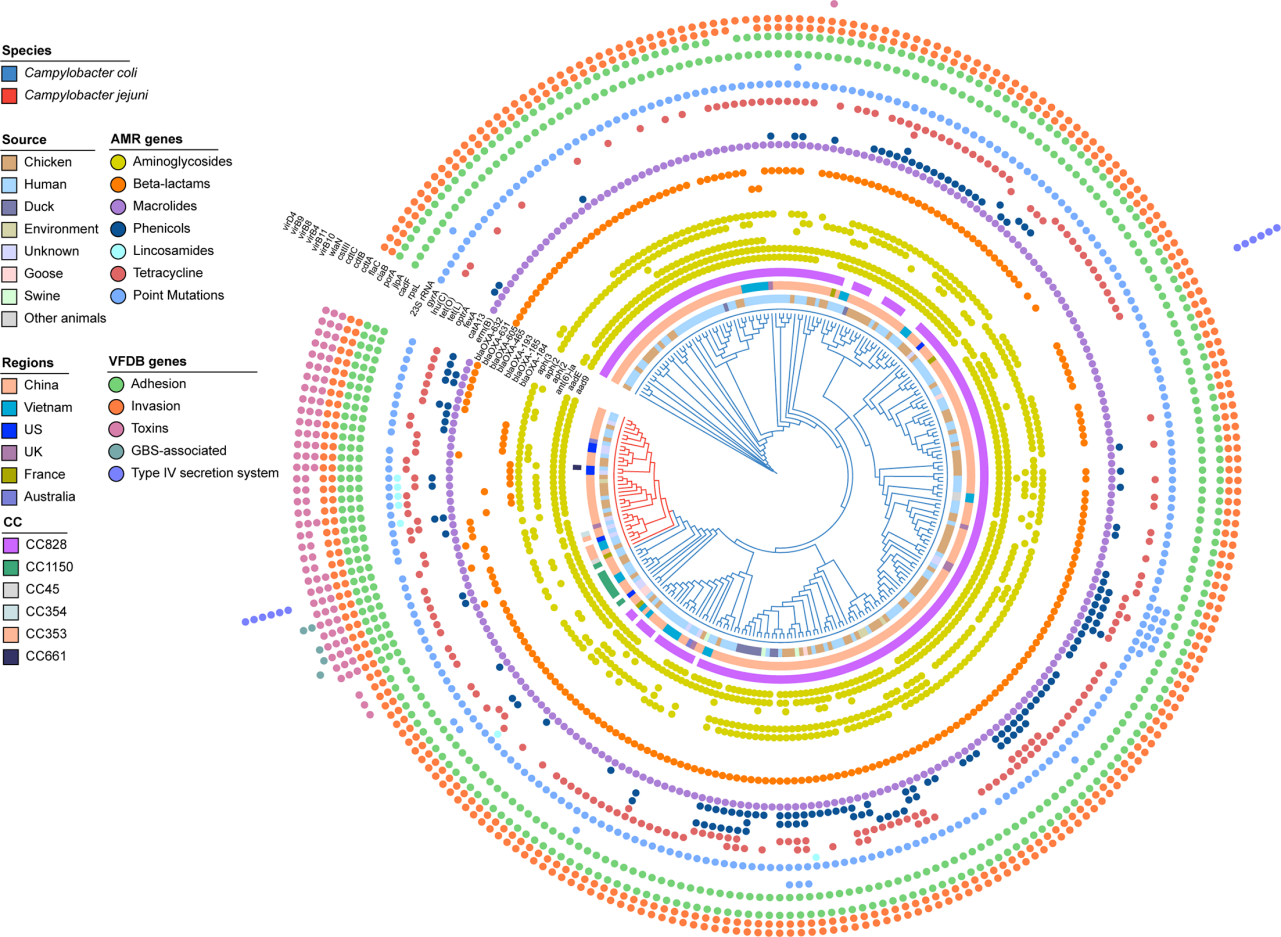
AMR profiles of 259 *Campylobacter* isolates based on a cladogram. Branch colour indicates species (*C. jejuni* and *C. coli*). From the centre, concentric circles indicate isolate sources, countries of origin and CCs, antibiotic resistance genes, mutations and virulence genes.

The distribution of 24 AMR genes and mutations in *erm*B-positive isolates varied among species and CCs. Principal coordinate analysis results showed that the distribution of antimicrobial resistance genes (ARGs) among isolates from poultry and clinical sources exhibited a more similar pattern compared to isolates from other sources (Fig. 2d). *C. coli* and *C. jejuni* have similar levels of AMR, but on average, *C. jejuni* isolate genomes contain significantly more virulence genes (*P*<0.05) (Figs 3 and S1). Nearly all isolates (98.84%, 256/259) harboured the T86I point mutation in the *gyrA* gene (Fig. 3), conferring resistance to quinolones, consistent with previous studies [56,57]. Aminoglycoside resistance genes, including *aad9* and *aadE*, were common in *C. coli* and *C. jejuni* isolates, being observed in 92% (238/259) and 96% (249/259) of the isolates, respectively (Fig. 3). The *aph(3')-IIIa* gene was the most common aminoglycoside resistance determinant, present in 78% (201/259) of the isolates. For genes encoding beta-lactamases, *bla*_OXA-61_ and *bla*_OXA-184_ family genes were present in 83% (216/259) and 10% (27/259) of isolates, respectively. The *bla*_OXA-184_ gene was exclusively identified in *C. jejuni* isolates at a frequency of 79% (27/34). The *bla*_OXA-605_ gene was the predominant beta-lactamase gene in *C. coli* isolate genomes (92%, 207/225) and was present in 21% (7/34) of *C. jejuni* isolates. In addition to *erm*(B), other genomic variation associated with macrolide resistance included the A2075G point mutation in 23S rRNA, detected in 2% of *C. coli* isolates (6/259). Other resistance genes included the following: (i) the lincosamide resistance gene *lnu*(C), present in 7 isolates (2 *C*. *coli* and 5 * C*. *jejuni*); (ii) the chloramphenicol resistance gene *catA13,* in 36% (94/259) of isolates; and (iii) the florfenicol resistance genes *fexA* and *optrA*, present in 50 isolates (37 *C*. *coli* and 13 *C*. *jejuni*).

The genomes of the 259 *erm*(B)-positive *Campylobacter* displayed considerable variation in the presence of 16 putative virulence genes (Fig. 3). These included genes associated with adhesion (*cadF*, *jlpA* and *porA*), invasion (*ciaB* and *flaC*), toxins (*cdtA*, *cdtB* and *cdtC*), putative Guillain-Barré syndrome (GBS)-associated genes (*wlaN* and *cst*-III) and the type IV secretion system (T4SS, *virB11*, *virB10*, *virB9*, *virB8*, *virB4* and *virD4*). Overall, the prevalence of virulent genes was significantly higher in *C. jejuni* than in *C. coli* isolates (*P*<0.05) (Fig. S1). Specifically, the adhesion-associated gene *jlpA* was present in all *C. jejuni* isolates but absent in all *C. coli* isolates. A similar trend was observed for the cytolethal distending toxin-associated genes (*cdtA*, *cdtB* and *cdtC*). Invasion-associated genes were prevalent in both *C. jejuni* and *C. coli*, with no significant difference (*P*>0.05). The GBS-associated genes *wlaN* or *cst-III* were detected in 4 *C*. *jejuni* isolates belonging to CC353, with one isolate harbouring both genes. The T4SS gene cluster was rare in *Campylobacter*, detected only in one *C. coli* and one *C. jejuni* isolate.

### The presence of *erm*(B) covaries with other pangenome elements

**Fig. 4.**
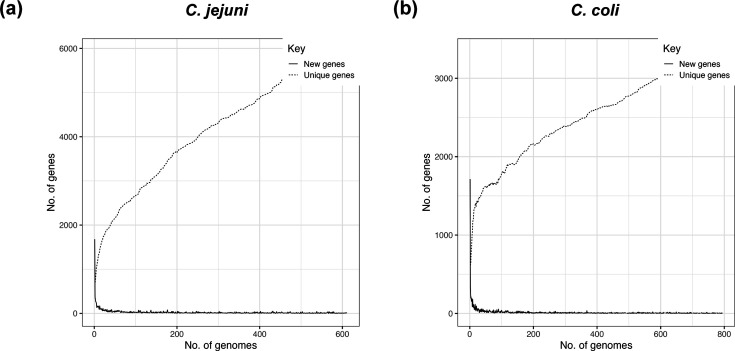
Roary pangenome plots illustrating relationships between numbers of identified genes and the number of *Campylobacter* genomes. (**a**) Cumulative number of genes plotted against the number of *C. jejuni* genomes. (**b**) Cumulative number of genes plotted against the number of *C. coli* genomes.

Pangenome analysis indicated that the increase in new gene discovery reached saturation as more genomes were included (Fig. 4). This demonstrated that among the samples in this study, a relatively stable pangenome was achieved from 611 * C*. *jejuni* (Fig. 4a) and 794 *C*. *coli* (Fig. 4b) genomes. To investigate potential functional variation associated with the presence of the *erm*(B) gene, we conducted a pangenome comparison among *erm*(B)-positive and *erm*(B)-negative isolates. Significant gene associations were identified at a *P*-value threshold of 0.05, with specificity for the absence of associated genes in trait-negative isolates set above 90%. For *C. jejuni*, 155 genes were significantly more common among isolates harbouring *erm*(B), including 52 with annotated function and 103 encoding hypothetical proteins. In *C. coli*, 97 genes with annotated function were more common among *erm*(B)-positive isolates and 387 genes encoding hypothetical proteins. While the chronology of gene acquisition and lineage (CC) expansion may influence this, 18 genes were differentially present in *erm*(B)-positive/negative isolates from both *C. jejuni* and *C. coli* (Table 1). This may be indicative of potentiation, co-acquisition and/or compensation following HGT [54].

**Table 1.** Functional annotations and prevalence of identified accessory elements associated with *erm*(B) in *Campylobacter*

Functional annotation	Gene	*C. jejuni*		*C. coli*	
		Sensitivity (%)	Specificity (%)	Sensitivity (%)	Specificity (%)
Antimicrobial genes	*tet*(L)	47.1	99.5	16.9	100
	*fexA*	23.5	99.5	12.9	100
	*aad9*	82.3	99.0	88	99.5
	*cat*	11.8	99.5	54.7	98.8
	*aadE*	100	95.0	92.9	99.3
	*aphA*	88.2	95.0	76	98.1
	*aacA/aphD*	52.9	97.4	81.8	99.8
	hph	67.6	97.2	27.6	98.2
Toxin–antitoxin system	Antitoxin epsilon	76.5	100	66.7	100
Transposases	IS1595 family ISBath1	20.6	97.2	83.1	100
	IS1595 family ISAcsp6	29.4	97.1	27.6	94.2
	IS1595 family ISCaje6	20.6	97.1	82.7	100
	IS607 family ISChh1	23.5	91.3	28.9	94.4
	IS21 family IS232	23.5	97.4	86.2	100
aa transport and metabolism	*mhpE*	8.8	99.7	12.4	98.2
Cell wall/membrane/envelope biogenesis	*neuA*	11.8	99.7	13.8	92.4
Coenzyme transport and metabolism	*tarD*	14.7	100	34.7	94.7
NAD(P)H-dependent oxidoreductase	*ywrO*	23.5	99.8	12	100
Total genes	18				

Multiple resistance genes, including *aadE*, *aphA3*, *aacA/aphD*, *tet*(L) and *catA*, were identified as more prevalent among *erm*(B)-positive isolates compared to susceptible isolates, potentially derived from Gram-positive bacteria such as *Staphylococcus*, *Streptococcus*, *Enterococcus* and *Bacillus* [10,17, 19]. This implies that the gut environment, where *Campylobacter* coexists with Gram-positive microbes, may facilitate the selection of *erm*(B) harbouring MDRGI. Varying ARG prevalence with the diverse MDRGIs is consistent with observations describing the genetic organization of ORFs, even though the *erm*(B) sequence remains conserved [10,17, 46, 58, 59].

**Fig. 5.**
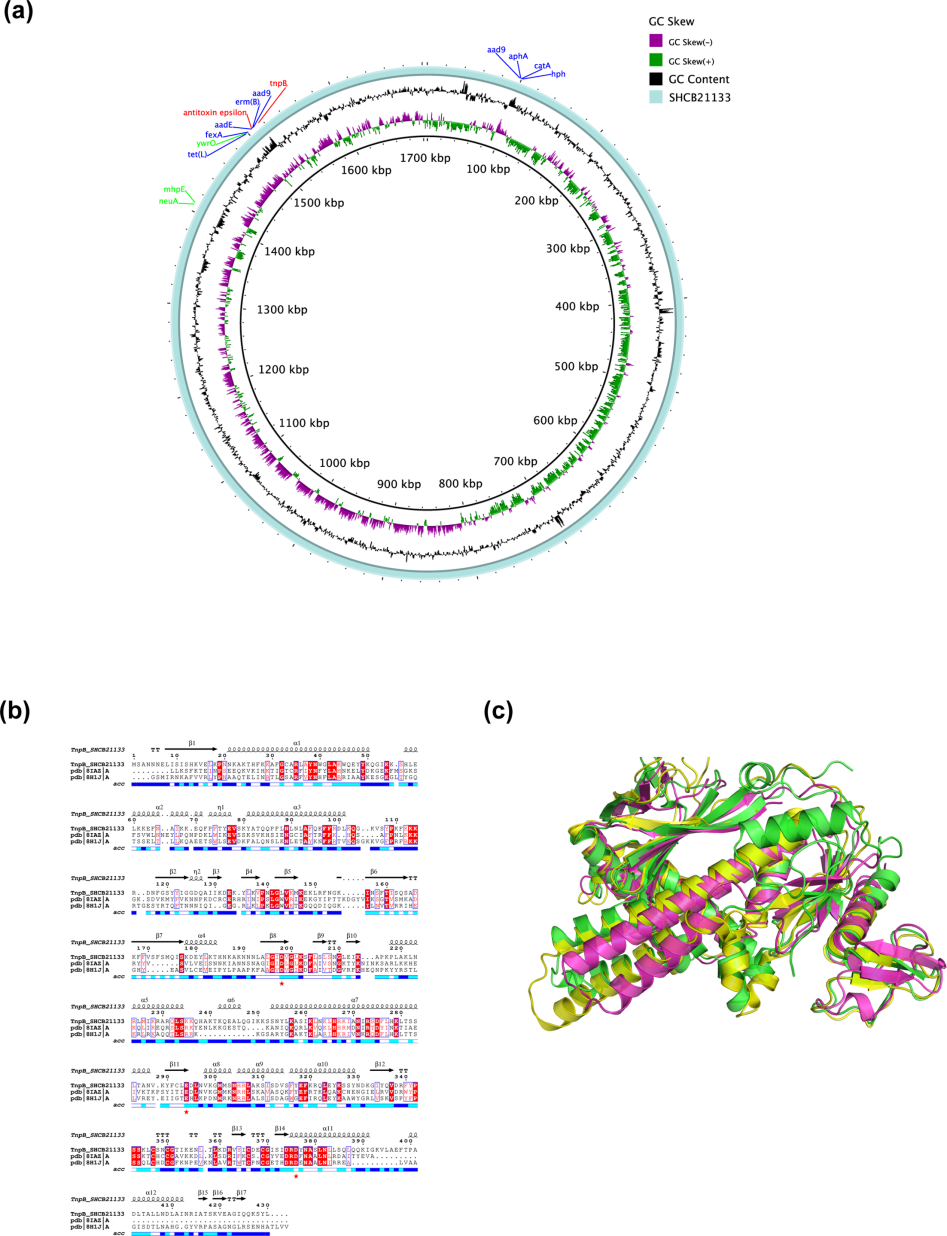
Potential functional linkage between TnpB and *erm*(B). (**a**) Genomic visualization of chromosomal accessory elements on the genome of *erm*(B)-carrying *C. jejuni* SHCB21133. GC skew, GC content and annotated genes are indicated from the inner to outer circle. (**b**) Protein sequence alignment of SHCB21133 TnpB and homologous proteins ISDra2 TnpB (pdb code: 8H1J) and ISFba1 TnpB (pdb code: 8IAZ); both were confirmed to possess DNase activity. Secondary structure elements are displayed at the top of each block of sequences. Relative accessibility is visualized as blue-coloured boxes at the bottom, with blue, cyan and white representing accessible, intermediate and buried, respectively. Similarity global score threshold was set to the default value of 0.7. Residues with scores above this threshold are displayed as coloured characters with blue frames, red characters on a white background by default or white characters on a red background for strictly conserved residues in the column. The conserved aa involved in the RuvC active site are marked with red stars. The sequence of ISFba1 TnpB (pdb: 8IAZ, D371A catalytic mutant) was reverted to WT. (**c**) Modelled structure of SHCB21133 TnpB superimposed onto homologous proteins with confirmed DNase activity (pdb: 8IAZ and 8H1J). SHCB21133 TnpB is shown in green, ISFba1 TnpB in yellow and ISDra2 TnpB in magenta.

Notably, the genes encoding toxin–antitoxin (TA) system were found exclusively in *erm*(B)-positive *Campylobacter* isolates with 100% specificity. blastp analysis showed a 99% aa identity to the homologue in *Streptococcus pyogenes* (Q57231.3), implying a possible Gram-positive bacterial origin. Moreover, several TEs covaried with the presence of *erm*(B), suggesting a potential link to the horizontal transfer of *erm*(B). Various other accessory elements commonly found in the genomes of *erm*(B)-positive strains were identified in the representative *C. jejuni* strain SHCB21133, including *aadE*, *aad9*, *aphA3*, *aacA/aphD*, *tet*(L)*, ywrO*, *fexA*, *hph*, *neuA* and *cat*, and antitoxin element, IS*Chh1 tnpB* (Fig. 5a) [46].

## Discussion

### The growing threat of AMR in *Campylobacter*

The rise of antimicrobial-resistant *Campylobacter* poses a critical global health challenge, severely constraining therapeutic strategies against this leading cause of bacterial gastroenteritis [1,2, 5]. Of particular concern is the emergence of *erm*(B)-mediated macrolide resistance, which has been increasingly documented across *Campylobacter* isolates from diverse ecological reservoirs [60,61]. However, the dissemination of *erm*(B)-carrying lineages in human infections and other sources remains unclear. The expanding global repository of bacterial genomic data facilitates systematic *in silico* exploration of *erm*(B)-carrying isolates and their associated genetic elements in antimicrobial-resistant *Campylobacter*. Here, we applied genomic approaches to analyse a comprehensive collection of *erm*(B)-positive clinical isolates from Shanghai, alongside a globally representative dataset encompassing all publicly available strains spanning diverse temporal and geographic origins.

### Poultry as a major reservoir for clinical *erm*(B)-positive *Campylobacter* isolates

Our analysis identified human and poultry as two primary reservoirs of *erm*(B)-harbouring *Campylobacter* isolates worldwide, similar to previous studies that have highlighted these sources as important reservoirs for antimicrobial-resistant *Campylobacter* [11,60, 62]. Phylogenetic analysis and pairwise comparisons of core and accessory genomes revealed a close relationship between *C. coli* isolates carrying the *erm*(B) gene from human and poultry sources. Additionally, four *erm*(B)-carrying *C. jejuni* isolates from both human and poultry origins clustered within poultry-associated CC CC353. The close phylogenetic proximity and clustering observed in the pangenome-sharing analysis further support poultry as the main source of *erm*(B)-positive *Campylobacter* infections in humans. While *erm*(B) was first reported in *C. coli* strain ZC113 of swine origin, it has since been predominantly isolated from poultry [10,59]. The reasons behind this shift, such as differences in sample coverage, host ecology, transmission dynamics or selective pressures, require further investigation and continuous monitoring to better understand the drivers of this trend.

### Differences in AMR between *C. coli* **and *****C. jejuni***

Antimicrobial-resistant *C. coli* isolates were previously reported to be better adapted than *C. jejuni* to the poultry industry in China, which may account for the shift in *C. coli* prevalence [60]. We also observed significantly higher macrolide resistance in *C. coli* than that in *C. jejuni* [9,60], potentially contributing to an increase in *C. coli* cases, rising from 10.6% during 2012–2016 to 19.0% during 2020–2023 [7]. While *erm*(B)-positive isolates were predominantly from *C. coli*, in line with recent studies [13,15, 16, 18, 58], the AMR comparison of *erm*(B)-positive isolates revealed no significant differences in the number of AMR genes between *C. coli* and *C. jejuni*. This observation contrasts with earlier studies that reported *C. coli* genomes harbouring more AMR genes than those of *C. jejuni* [45,63]. The discrepancy may be due to differences in isolate sources, as our study specifically focused on *erm*(B)-positive isolates rather than the broader population of antimicrobial-resistant *Campylobacter*.

### Associated AMR elements in *erm*(B)-positive isolates

Pangenome analysis further supports this observation, revealing multiple AMR genes associated with the presence of *erm*(B) in both *C. jejuni* and *C. coli*. There is evidence of the transfer of *erm*(B)-carrying MDRGIs between *C. coli* and *C. jejuni* [16,46], which could have led to the simultaneous acquisition of multiple AMR genes. The co-occurrence of AMR genes with *erm*(B), particularly derived from Gram-positive bacteria, highlights the circulation of AMR determinants not only among *Campylobacter* species in different habitats but also through HGT from other bacterial taxa [16,64, 65]. Additionally, the TA system identified in both *C. coli* and *C. jejuni* appears to be a common feature within MDRGIs [17]. TA systems, which vary widely in function and composition across organisms, are increasingly recognized as bacterial defence mechanisms against antimicrobial agents [66,67]. These systems are more frequently found in organisms exposed to stressful or hostile environments, where HGT is common, and serve as strategies for bacterial survival [68,70]. Several TA systems have been characterized in *Campylobacter* [66,70], and the TA system identified in this study shares homology with one in *S. pyogenes*, known for its role in plasmid maintenance [71]. The exclusive presence of this TA system in *erm*(B)-positive isolates suggests its involvement in promoting the stability and transmission of the *erm*(B) gene under antimicrobial pressure.

### Association of *erm*(B) with transposons and potential links to bacterial immunity

Several AMR genes have been previously found to be located in proximity to transposase genes, facilitating the acquisition of these resistance genes [72,73]. Pangenome analysis revealed that *erm*(B) was associated with multiple transposases in both * C. jejuni* and *C. coli*. Further sequence and structural analysis of the *erm*(B)-positive *C. jejuni* strain SHCB21133 identified the first *erm*(B)-associated transposon protein TnpB in *C. jejuni*. This TnpB protein exhibited homology to TnpB proteins from IS200/IS600 and IS607 transposon families [74,75], revealing high secondary structural similarity and conserved domain organization (Fig. 5b), with aa identities of 34% and 33%, respectively. The transposon-encoded TnpB protein is predicted to be an ancestor of the Cas12 nucleases found in CRISPR-Cas systems [74,76], which are part of bacterial defence systems that provide adaptive immunity and have already been utilized for genome editing. Conserved residues D191, E278 and D361, critical to the catalytic centre of the RuvC domain in ISDra2 TnpB, are also present in SHCB21133 ISChh1 TnpB at corresponding positions D198, E294 and D376 (Fig. 5b). Structural comparison between the modelled TnpB of SHCB21133 and thecryogenic electron microscopy structures of the ISDra2 TnpB and ISFba1 TnpB displayed similar 3D architecture, with root mean square deviation values of 2.727 and 1.461 Å, respectively, suggesting potential similarities in catalytic activity (Fig. 5c). Previous studies have also identified a novel *erm*N gene located exclusively within CRISPR arrays in *C. coli* [15,77], highlighting an intriguing link between the macrolide resistance determinants and bacterial immunity systems. Furthermore, the *tnpB* gene identified within the accessory elements of the SHCB21133 genome exhibited a 99.8% identity with the previously reported ISChh1-like element of optrA-containing fragment (MT780492). This transposon belongs to the IS607 family, which mediates the acquisition of the *optrA* gene in *C. coli* [73]. Further investigation, including functional studies such as TnpB gene knockout experiments, is required to clarify the intricate relationship between *erm* genes and the bacterial immunity system, which may shed light on their role in bacterial adaptation and horizontal gene acquisition.

In conclusion, the widespread use of antibiotics, particularly in clinical and poultry settings, may have provided the antibiotic pressure promoting the global dissemination of *erm*(B)-positive *Campylobacter* isolates, with a notably higher prevalence observed in poultry from China. Our study underscores the significant role of poultry as a reservoir for *erm*(B) in clinical cases. The rational use of antimicrobials in poultry industries could effectively reduce the selective fitness of *Campylobacter* isolates and mitigate the risk of human infections. Also, multiple accessory genes are associated with the *erm*(B) acquisition in both *C. jejuni* and * C. coli*. Continuous global genomic surveillance of *erm*(B)-positive *Campylobacter* is crucial for tracking its transmission dynamics and curbing its dissemination.

## Supplementary material

10.1099/mgen.0.001528 Uncited Supplementary Material 1.

10.1099/mgen.0.001528 Uncited Table S1.

10.1099/mgen.0.001528 Uncited Table S2.

10.1099/mgen.0.001528 Uncited Table S3.

## Abbreviations

AMR: antimicrobial resistance
ANI: average nt identity
CC: clonal complex
HGT: horizontal gene transfer
MDRGI: multidrug resistance genomic island
MICs: minimum inhibitory concentrations
ST: sequence type
TA: toxin–antitoxin
TE: transposable element
T4SS: type IV secretion system
VFDB: Virulence Factor Database

## References

[1] Kaakoush NO, Castaño-Rodríguez N, Mitchell HM, Man SM (2015). Global Epidemiology of Campylobacter Infection. Clin Microbiol Rev.

[2] European Food Safety Authority (EFSA), European Centre for Disease Prevention and Control (ECDC) The european union summary report on antimicrobial resistance in zoonotic and indicator bacteria from humans, animals and food in 2021–2022. EFS2.

[3] Scallan E, Hoekstra RM, Mahon BE, Jones TF, Griffin PM (2015). An assessment of the human health impact of seven leading foodborne pathogens in the United States using disability adjusted life years. Epidemiol Infect.

[4] CDC (2019). Antibiotic Resistance Threats in the United States, 2019.

[5] Mourkas E, Florez-Cuadrado D, Pascoe B, Calland JK, Bayliss SC (2019). Gene pool transmission of multidrug resistance among *Campylobacter* from livestock, sewage and human disease. Environ Microbiol.

[6] Zhang P, Zhang X, Liu Y, Jiang J, Shen Z (2017). Multilocus sequence types and antimicrobial resistance of *Campylobacter jejuni* and *C. coli* isolates of human patients from Beijing, China, 2017–2018. Front Microbiol.

[7] Gao F, Tu L, Chen M, Chen H, Zhang X (2023). Erythromycin resistance of clinical *Campylobacter jejuni* and *Campylobacter coli* in Shanghai, China. Front Microbiol.

[8] Zhao Q, Jiang Z, Li T, Cheng M, Sun H (2023). Current status and trends in antimicrobial use in food animals in China, 2018–2020. *One Health Adv*.

[9] Zhou J, Zhang M, Yang W, Fang Y, Wang G (2016). A seventeen-year observation of the antimicrobial susceptibility of clinical *Campylobacter jejuni* and the molecular mechanisms of erythromycin-resistant isolates in Beijing, China. Int J Infect Dis.

[10] Qin S, Wang Y, Zhang Q, Zhang M, Deng F (2014). Report of ribosomal RNA methylase gene erm(B) in multidrug-resistant *Campylobacter coli*. J Antimicrob Chemother.

[11] Liu P, Qin X, Cao T, Yang Y, Shi X (2022). Telithromycin resistance in *Campylobacter* mediated by 23S rRNA A2075G mutation and erm(B). J Antimicrob Chemother.

[12] Chen JC, Tagg KA, Joung YJ, Bennett C, Francois Watkins L (2018). Report of erm (b) + campylobacter jejuni in the united states. Antimicrob Agents Chemother.

[13] Florez-Cuadrado D, Ugarte-Ruiz M, Meric G, Quesada A, Porrero MC (2017). Genome comparison of erythromycin resistant *Campylobacter* from Turkeys identifies hosts and pathways for horizontal spread of *erm*(B) genes. Front Microbiol.

[14] Elhadidy M, Miller WG, Arguello H, Álvarez-Ordóñez A, Dierick K (2019). Molecular epidemiology and antimicrobial resistance mechanisms of Campylobacter coli from diarrhoeal patients and broiler carcasses in Belgium. Transbound Emerg Dis.

[15] Jehanne Q, Bénéjat L, Ducournau A, Domingues-Martins C, Cousinou T (2021). Emergence of erythromycin resistance methyltransferases in *Campylobacter coli* strains in France. Antimicrob Agents Chemother.

[16] Liu D, Liu W, Lv Z, Xia J, Li X (2019). Emerging *erm*(B)-mediated macrolide resistance associated with novel multidrug resistance genomic Islands in *Campylobacter*. Antimicrob Agents Chemother.

[17] Shen Z, Wang Y, Zhang Q, Shen J (2018). Antimicrobial resistance in *Campylobacter spp*. Microbiol Spectr.

[18] Wang Y, Zhang M, Deng F, Shen Z, Wu C (2014). Emergence of multidrug-resistant *Campylobacter* species isolates with a horizontally acquired rRNA methylase. Antimicrob Agents Chemother.

[19] Bolinger H, Kathariou S (2017). The current state of macrolide resistance in *Campylobacter spp*.: trends and impacts of resistance mechanisms. Appl Environ Microbiol.

[20] Chen J, Yu Z, Michel FC, Wittum T, Morrison M (2007). Development and application of real-time PCR assays for quantification of *erm* genes conferring resistance to macrolides-lincosamides-streptogramin B in livestock manure and manure management systems. Appl Environ Microbiol.

[21] Bankevich A, Nurk S, Antipov D, Gurevich AA, Dvorkin M (2012). SPAdes: a new genome assembly algorithm and its applications to single-cell sequencing. J Comput Biol.

[22] Parks DH, Imelfort M, Skennerton CT, Hugenholtz P, Tyson GW (2015). CheckM: assessing the quality of microbial genomes recovered from isolates, single cells, and metagenomes. Genome Res.

[23] Jolley KA, Maiden MCJ (2010). BIGSdb: scalable analysis of bacterial genome variation at the population level. BMC Bioinformatics.

[24] Jolley KA, Bray JE, Maiden MCJ (2018). Open-access bacterial population genomics: BIGSdb software, the PubMLST.org website and their applications. Wellcome Open Res.

[25] Dingle KE, Colles FM, Wareing DR, Ure R, Fox AJ (2001). Multilocus sequence typing system for *Campylobacter jejuni*. J Clin Microbiol.

[26] Center for Disease Control Prevention [CDC] National Antimicrobial Resistance Monitoring System for Enteric Bacteria (NARMS). Antibiotics Tested by NARMS. https://www.cdc.gov/narms/about/antibiotics-tested.html.

[27] Feldgarden M, Brover V, Haft DH, Prasad AB, Slotta DJ (2019). Validating the AMRfinder tool and resistance gene database by using antimicrobial resistance genotype-phenotype correlations in a collection of isolates. Antimicrob Agents Chemother.

[28] Chen L, Zheng D, Liu B, Yang J, Jin Q (2016). VFDB 2016: hierarchical and refined dataset for big data analysis--10 years on. Nucleic Acids Res.

[29] Zankari E, Allesøe R, Joensen KG, Cavaco LM, Lund O (2017). PointFinder: a novel web tool for WGS-based detection of antimicrobial resistance associated with chromosomal point mutations in bacterial pathogens. J Antimicrob Chemother.

[30] Seemann T (2014). Prokka: rapid prokaryotic genome annotation. Bioinformatics.

[31] Bayliss SC, Thorpe HA, Coyle NM, Sheppard SK, Feil EJ (2019). PIRATE: a fast and scalable pangenomics toolbox for clustering diverged orthologues in bacteria. Gigascience.

[32] Jain C, Rodriguez-R LM, Phillippy AM, Konstantinidis KT, Aluru S (2018). High throughput ANI analysis of 90K prokaryotic genomes reveals clear species boundaries. Nat Commun.

[33] Sheppard SK, Jolley KA, Maiden MCJ (2012). A gene-by-gene approach to bacterial population genomics: whole genome MLST of *Campylobacter*. Genes.

[34] Price MN, Dehal PS, Arkin AP (2010). FastTree 2--approximately maximum-likelihood trees for large alignments. PLoS One.

[35] Argimón S, Abudahab K, Goater RJE, Fedosejev A, Bhai J (2016). Microreact: visualizing and sharing data for genomic epidemiology and phylogeography. Microbial Genom.

[36] Letunic I, Bork P (2007). Interactive Tree Of Life (iTOL): an online tool for phylogenetic tree display and annotation. Bioinformatics.

[37] Page AJ, Cummins CA, Hunt M, Wong VK, Reuter S (2015). Roary: rapid large-scale prokaryote pan genome analysis. Bioinformatics.

[38] Brynildsrud O, Bohlin J, Scheffer L, Eldholm V (2016). Rapid scoring of genes in microbial pan-genome-wide association studies with Scoary. Genome Biol.

[39] McGinnis S, Madden TL (2004). BLAST: at the core of a powerful and diverse set of sequence analysis tools. Nucleic Acids Res.

[40] Papadopoulos JS, Agarwala R (2007). COBALT: constraint-based alignment tool for multiple protein sequences. Bioinformatics.

[41] Robert X, Gouet P (2014). Deciphering key features in protein structures with the new ENDscript server. Nucleic Acids Res.

[42] Varadi M, Bertoni D, Magana P, Paramval U, Pidruchna I (2024). Alphafold protein structure database in 2024: providing structure coverage for over 214 million protein sequences. Nucleic Acids Res.

[43] Jumper J, Evans R, Pritzel A, Green T, Figurnov M (2021). Highly accurate protein structure prediction with alphafold. Nature.

[44] Schrödinger L, DeLano W (2020). PyMOL. http://www.pymol.org/pymol.

[45] Liao Y-S, Chen B-H, Teng R-H, Wang Y-W, Chang J-H (2022). Antimicrobial resistance in *Campylobacter coli* and *Campylobacter jejuni* from human campylobacteriosis in Taiwan, 2016 to 2019. Antimicrob Agents Chemother.

[46] Gao F, Luo J, Chen M (2024). Characterization of erm(B) in a clinical *Campylobacter jejuni* isolate from China. J Antimicrob Chemother.

[47] Dearlove BL, Cody AJ, Pascoe B, Méric G, Wilson DJ (2016). Rapid host switching in generalist *Campylobacter* strains erodes the signal for tracing human infections. ISME J.

[48] Sheppard SK, Colles FM, McCarthy ND, Strachan NJC, Ogden ID (2011). Niche segregation and genetic structure of *Campylobacter jejuni* populations from wild and agricultural host species. Mol Ecol.

[49] Sheppard SK, Colles F, Richardson J, Cody AJ, Elson R (2010). Host association of *Campylobacter* genotypes transcends geographic variation. Appl Environ Microbiol.

[50] Sheppard SK, Cheng L, Méric G, de Haan CPA, Llarena A-K (2014). Cryptic ecology among host generalist *Campylobacter jejuni* in domestic animals. Mol Ecol.

[51] Pascoe B, Futcher G, Pensar J, Bayliss SC, Mourkas E (2024). Machine learning to attribute the source of *Campylobacter* infections in the United States: a retrospective analysis of national surveillance data. J Infect.

[52] Sheppard SK, Dallas JF, MacRae M, McCarthy ND, Sproston EL (2009). *Campylobacter* genotypes from food animals, environmental sources and clinical disease in Scotland 2005/6. Int J Food Microbiol.

[53] Sheppard SK, McCarthy ND, Falush D, Maiden MCJ (2008). Convergence of *Campylobacter* species: implications for bacterial evolution. Science.

[54] Taylor AJ, Yahara K, Pascoe B, Ko S, Mageiros L (2024). Epistasis, core-genome disharmony, and adaptation in recombining bacteria. *mBio*.

[55] Sheppard SK, Didelot X, Jolley KA, Darling AE, Pascoe B (2013). Progressive genome-wide introgression in agricultural *Campylobacter coli*. Mol Ecol.

[56] Wimalarathna HML, Richardson JF, Lawson AJ, Elson R, Meldrum R (2013). Widespread acquisition of antimicrobial resistance among *Campylobacter* isolates from UK retail poultry and evidence for clonal expansion of resistant lineages. BMC Microbiol.

[57] Sproston EL, Wimalarathna HML, Sheppard SK (2018). Trends in fluoroquinolone resistance in *Campylobacter*. *Microb Genom*.

[58] Florez-Cuadrado D, Ugarte-Ruiz M, Quesada A, Palomo G, Domínguez L (2016). Description of an erm(B)-carrying *Campylobacter coli* isolate in Europe. J Antimicrob Chemother.

[59] Liu D, Deng F, Gao Y, Yao H, Shen Z (2017). Dissemination of erm(B) and its associated multidrug-resistance genomic islands in *Campylobacter* from 2013 to 2015. Vet Microbiol.

[60] Zhang P, Zhang X, Liu Y, Cui Q, Qin X (2022). Genomic insights into the increased occurrence of campylobacteriosis caused by antimicrobial-resistant *Campylobacter coli*. mBio.

[61] Qin X, Wang X, Shen Z (2023). The rise of antibiotic resistance in *Campylobacter*. Curr Opin Gastroenterol.

[62] Li X, Xu X, Chen X, Li Y, Guo J (2023). Prevalence and genetic characterization of *Campylobacter* from clinical poultry cases in China. Microbiol Spectr.

[63] Zhang A, Song L, Liang H, Gu Y, Zhang C (2016). Molecular subtyping and erythromycin resistance of campylobacter in China. J Appl Microbiol.

[64] Tang B, Tang Y, Zhang L, Liu X, Chang J (2020). Emergence of *fexA* in mediating resistance to florfenicols in *Campylobacter*. Antimicrob Agents Chemother.

[65] Li W, Jiao D, Kang J, Yu R, Zhao W (2023). Emergence of lnu(C) variant conferring lincomycin resistance in *Campylobacter coli* of chicken origin. Int J Food Microbiol.

[66] Wadie B, Abdel-Fattah MA, Yousef A, Mouftah SF, Elhadidy M (2021). In silico characterization of toxin-antitoxin systems in *Campylobacter* isolates recovered from food sources and sporadic human illness. Genes.

[67] Schuster CF, Bertram R (2016). Toxin-antitoxin systems of *Staphylococcus aureus*. Toxins.

[68] Jurėnas D, Fraikin N, Goormaghtigh F, Van Melderen L (2022). Biology and evolution of bacterial toxin-antitoxin systems. Nat Rev Microbiol.

[69] Chen Y, Goh Y-X, Li P, Guan J, Chao Y (2024). RES-Xre toxin-antitoxin locus *knaAT* maintains the stability of the virulence plasmid in *Klebsiella pneumoniae*. Emerg Microbes Infects.

[70] Shen Z, Patil RD, Sahin O, Wu Z, Pu X-Y (2016). Identification and functional analysis of two toxin-antitoxin systems in *Campylobacter jejuni*. Mol Microbiol.

[71] Meinhart A, Alonso JC, Sträter N, Saenger W (2003). Crystal structure of the plasmid maintenance system epsilon/zeta: functional mechanism of toxin zeta and inactivation by epsilon 2 zeta 2 complex formation. Proc Natl Acad Sci USA.

[72] Zarske M, Luu HQ, Deneke C, Knüver M-T, Thieck M (2024). Identification of knowledge gaps in whole-genome sequence analysis of multi-resistant thermotolerant Campylobacter spp. BMC Genomics.

[73] Tang Y, Lai Y, Wang X, Lei C, Li C (2021). Novel insertion sequence ISChh1-like mediating acquisition of optrA gene in foodborne pathogen Campylobacter coli of swine origin. Vet Microbiol.

[74] Nakagawa R, Hirano H, Omura SN, Nety S, Kannan S (2023). Cryo-EM structure of the transposon-associated TnpB enzyme. Nature.

[75] Ren K, Zhou F, Zhang F, Yin M, Zhu Y (2024). Discovery and structural mechanism of DNA endonucleases guided by RAGATH-18-derived RNAs. Cell Res.

[76] Makarova KS, Wolf YI, Iranzo J, Shmakov SA, Alkhnbashi OS (2020). Evolutionary classification of CRISPR-Cas systems: a burst of class 2 and derived variants. Nat Rev Microbiol.

[77] Greninger AL, Addetia A, Starr K, Cybulski RJ, Stewart MK (2020). International spread of multidrug-resistant *Campylobacter coli* in men who have sex with men in Washington State and Québec, 2015–2018. Clin Infect Dis.

