# Supplementary material for: Genomic epidemiology and the evolution of erm(B)-mediated macrolide resistance in Campylobacter
Source: Microb Genom. 2025 Oct 8;11(10):001528. doi: 10.1099/mgen.0.001528 (PMC12507524; doi:10.1099/mgen.0.001528)
Supplement: Uncited Supplementary Material 1. [file mgen-11-01528-s001.pdf]

Supplementary Materials for

Genomic epidemiology and the evolution of *erm*(B)-mediated macrolide resistance in *Campylobacter*

Fen Gao[a], Frances M. Colles[b], Seungwon Ko[b], Jiayuan Luo[a], Samuel K. Sheppard[b]#, Min Chen[a]#

[a]Department of Microbiology, Shanghai Municipal Center for Disease Control and Prevention, Shanghai, China

[b]Department of Biology, University of Oxford, Oxford, UK

#Address correspondence to Samuel K. Sheppard, samuel.sheppard@biology.ox.ac.uk, or Min Chen, chenmin@scdc.sh.cn.

This file includes:

Fig. S1

Other Supplementary Materials for this manuscript include the following:

Table S1 to S3

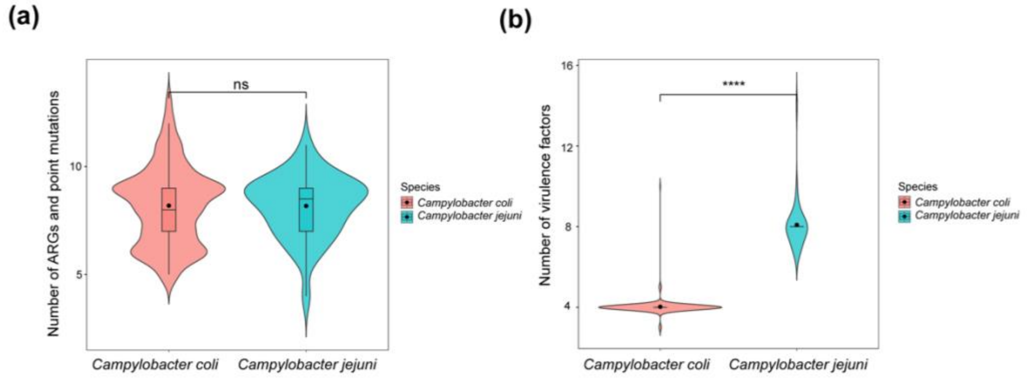

Fig. S1. Prevalence of putative antimicrobial-resistance and virulence genes among *erm*(B)-positive isolates. (a) The numbers of antibiotic resistance genes (ARGs) and point mutations identified in *C. jejuni* and *C. coli* isolate genomes. (b) The numbers of virulence factors detected in *C. jejuni* and *C. coli* isolate genomes. The horizontal bar represents the median, while the black dot indicates the average value. The p-values in both panels were calculated using the Wilcoxon signed-rank test for significant difference. Statistical significance is denoted by four asterisks for $P \leq 0.0001$, while ns indicates no significant difference ($P > 0.05$).

Table S1. (separate file) List of isolates carrying the *erm*(B) gene used in this study.

Table S2. (separate file) *C. jejuni* isolates used for pangenome-wide association analysis.

Table S3. (separate file) *C. coli* isolates used for pangenome-wide association analysis.
